# Assessing academic entitlement in pharmacy students: insights from Saudi Arabia

**DOI:** 10.3389/fmed.2025.1553233

**Published:** 2025-07-09

**Authors:** Dalia Almaghaslah, Bayan Alamri

**Affiliations:** ^1^Department of Clinical Pharmacy, College of Pharmacy, King Khalid University, Abha, Saudi Arabia; ^2^College of Pharmacy, King Khalid University, Abha, Saudi Arabia

**Keywords:** academic entitlement (AE), pharmacy students, Saudi Arabia, consumerism, Pharm D student

## Abstract

**Background:**

Academic entitlement, characterized by students’ expectations of academic rewards without proportional effort, has become an emerging concern in higher education, particularly in pharmacy programs. Understanding the demographic factors contributing to this phenomenon can help institutions design targeted interventions to mitigate its effects.

**Aim:**

The aim of this study was to assess Academic Entitlement (AE) its seven subscales, including Rewards for Effort, Accommodation, Responsibility Avoidance, Customer Orientation, Customer Service Expectation, and Grade Haggling among pharmacy students in Saudi Arabia. Also to assess association between AE and demographics including age, gender and GPA.

**Methods:**

A 17-item Academic Entitlement Questionnaire was distributed to pharmacy students through an online survey. The questionnaire, translated into Arabic using the back-translation method, was piloted for clarity before distribution. Data were analyzed using inferential statistics to assess the association between academic entitlement and key demographic variables.

**Results:**

A total of 267 pharmacy students enrolled in Pharm D program participated in the study. Statistical analysis revealed significant associations between academic entitlement and specific demographic factors: Age: A significant relationship was observed (*P* = 0.032), indicating variations in entitlement levels across age groups. Gender: No significant relationship was found (*P* = 0.242). GPA: No significant association was identified (*P* = 0.42), suggesting entitlement levels may vary with academic performance. These findings suggest that younger students may exhibit higher levels of academic entitlement.

**Conclusion:**

Academic entitlement among pharmacy students is influenced by demographic factors, with significant associations observed for age. These findings highlight the need for educational strategies that address entitlement behaviors, particularly among younger, to maintain academic rigor and professional accountability. Further research is needed to explore underlying causes and effective interventions.

## Introduction

The rise of academic entitlement presents a growing concern within higher education, with implications for both faculty and institutional leadership (1). Academic entitlement is characterized by the expectation of academic success without taking personal responsibility for the effort required to achieve it (2). Another definition describes academic entitlement as a student’s belief that they deserve higher grades or special treatment without putting in significant time or effort (3, 4).

Another term often used interchangeably with academic entitlement is student consumerism, which reflects the attitude that higher grades and academic achievements are justified simply because students have paid for their education (2, 5, 6).

A pharmacy degree is more than just an economic investment; however, the rising cost of tuition, decreasing affordability of higher education, and the significant time commitment involved have contributed to the growing perception of students as consumers (7). This mindset is further shaped by societal and cultural factors. The rise in academic entitlement has been linked to increasing generational narcissism, evolving educational paradigms, and the influence of technology and media. Students are often immersed in student-centered learning environments that emphasize constant positive reinforcement and, in some cases, unwarranted grade inflation. Additionally, the widespread use of self-promotional platforms like YouTube and Facebook—where self-glorification is commonplace—may reinforce entitled attitudes and unrealistic expectations (1, 2, 8).

General entitlement differs from academic entitlement in important ways. Individuals with a general sense of entitlement believe they deserve preferential treatment or outcomes simply because they perceive themselves as inherently superior to others (2, 5, 6).

Academic entitlement has been reported in pharmacy institutions. The concept of AE is reflected in attitudes that are translated into observable behaviors, including inappropriate use of technology during class, arriving late or leaving early, neglecting preparatory tasks, and minimal participation in group work (9, 10).

While academic entitlement has not been thoroughly investigated among pharmacy students. A recent study conducted in the United States found that pharmacy students view themselves as both consumers and products of their education—a dual perspective not previously reported. In contrast, faculty and preceptors perceived students solely as products of the educational process. This coexistence of views highlights the complexity of student perceptions in pharmacy education. Although students demonstrated high levels of academic entitlement, many still expressed a strong desire to become competent professionals. Nevertheless, such entitlement attitudes may be associated with unprofessional behaviors that can hinder their development into effective healthcare providers (11).

Another study is a multicounty study examined Arab pharmacy students’ responses across seven domains of academic entitlement. The findings indicated that students tended to endorse attitudes related to rewards for effort, customer orientation, customer service expectations, and general academic entitlement. These attitudes reflect students’ beliefs about their rights and expectations within the educational system (1). This suggests that academic entitlement remains high among pharmacy students despite cultural and social differences across study settings.

Academic entitlement has been reported across a wide range of disciplines beyond pharmacy, including psychology, business, physical therapy, and other health-related professional programs. Studies in these fields have consistently highlighted the presence of entitled attitudes among students, often characterized by unrealistic academic expectations, demands for preferential treatment, and resistance to constructive feedback. This widespread occurrence suggests that AE is a cross-disciplinary concern that can influence student behavior, academic integrity, and educator-student relationships in diverse educational settings (12–17).

Academic entitlement has several significant implications, including grade inflation, student incivility, and altered teaching practices. Grade inflation raises serious concerns about the effects of AE on the standards and rigor of pharmacy education. Many worry that grade inflation, driven by the demands of entitled students, could result in graduates lacking the necessary skills and attitudes to maintain the high quality of care and expertise expected by both the profession and the public (3).

Increased academic entitlement has been linked to a range of negative outcomes, including reduced engagement both inside and outside the classroom, poor social adjustment to university life, ineffective academic emotion regulation, and inappropriate classroom behaviors. Additionally, entitled students tend to show greater acceptance of plagiarism, academic dishonesty, and incivility. They are also more likely to perceive cheating as less unethical compared to their peers (5, 16, 18–20).

Incivility, which can manifest in behaviors such as loud sarcastic remarks, arguments with faculty, or tardiness, signals disrespect for the educational process. Students may exhibit such behaviors as a means to express power, frustration, or to gain something of value. AE may exacerbate these issues, as entitled students often exert pressure on faculty to accommodate their demands, such as digital lectures or detailed exam information. This consumer-driven mindset can negatively impact faculty morale, especially among junior faculty who may lack the experience to handle such pressures. Faculty may feel disrespected or undervalued, potentially leading to cynicism and strained relationships with students. Additionally, the rapid amplification of student demands through social media can escalate incivility and encourage peer support, further complicating the issue (3, 10, 21).

Pharmacy education is fundamentally designed to serve the needs of patients, positioning them—not students—as the central focus of the learning process. This patient-centered approach aligns with the principles of pharmaceutical care and reinforces the professional responsibility of future pharmacists. By viewing the patient as the primary beneficiary of education, academic entitlement and student consumerism may be reduced, while greater emphasis is placed on developing the knowledge, skills, and attitudes required to provide high-quality care (6).

In pharmacy education, academic entitlement is contrasted with a proposed model that emphasizes the responsibilities institutions must uphold. While entitlement may be present, it is essential that students are assured: (1) the opportunity to learn, (2) instruction by faculty committed to effective teaching practices, (3) a curriculum that prepares them for professional practice, and (4) access to the necessary resources for academic success (7).

Pharmacy curricula in Saudi Arabia and the United States share the goal of producing competent, patient-centered practitioners, yet they differ in structure and credit hour distribution. In Saudi Arabia, pharmacy programs require a minimum of 160 credit hours, with a strong emphasis on experiential training (33%), pharmacotherapy (16%), and clinical pharmacy sciences (15%). The remaining credits are distributed among biomedical sciences (11%), pharmaceutics (10%), pharmacology (7%), medicinal chemistry (6%), and pharmaceutical research (4%) (22). In contrast, United States pharmacy curricula allocate credits across broader categories, with averages of 10.6 credits in biomedical sciences, 25.3 in pharmaceutical sciences, 17.1 in social/administrative/behavioral sciences, 40.5 in clinical sciences, 45.5 in experiential education, and 7.0 in electives. Although both systems incorporate foundational and clinical components, United States programs show greater emphasis on clinical and experiential learning (23, 24).

The aim of this study was to assess General Academic Entitlement (AE) its seven subscales, including Rewards for Effort, Accommodation, Responsibility Avoidance, Customer Orientation, Customer Service Expectation, Grade Haggling among pharmacy students in Saudi Arabia. Also to assess association between AE and demographics including age, gender and GPA.

## Materials and methods

### Study design

This study utilized a cross-sectional design conducted between October 2024 and January 2025.

### Setting

The research was conducted at a College of Pharmacy in Saudi Arabia, targeting students enrolled in the Pharm D program at a Saudi government university.

### Participants

The study included pharmacy students from Year 1 to Year 5 and pharmacy interns aged 18 years or older. Students enrolled in other academic programs and those who refused to take part were excluded. This ensured that the results reflected only the views of current Pharm D students. Only participants who met the inclusion criteria and completed the questionnaire were included in the final analysis.

### Sample size and sampling procedure

A non-probability convenience sampling method was used to recruit participants. Invitations were initially sent via the official university email to all eligible Pharm D students (*N* = 820). To enhance participation, a reminder email was sent 2 weeks after the initial invitation, followed by a second reminder after 1 month. A total of 221 students responded via email. As no further increase in responses was observed after the second reminder, in-person recruitment was initiated within the college halls to boost participation.

The required sample size was calculated to be 262 using the Raosoft sample size calculator, based on a 5% margin of error and a 95% confidence level. This sample size was chosen to ensure adequate power and reliability in examining correlations between academic entitlement scores and various demographic variables, and to minimize potential sampling error.

### Data collection tool

The questionnaire consisted of two main sections. The first section collected demographic and background information, including age, gender, and GPA. The second section assessed academic entitlement through 17 items categorized into seven domains: rewards for effort (two items), accommodation (two items), responsibility avoidance (two items), customer orientation (two items), customer service expectations (three items), grade haggling (three items), and general academic entitlement (three items).

The 17-item Academic Entitlement Questionnaire (AEQ) used in this study was derived from the original scale developed by Jackson et al. (25), designed to measure students’ perceptions of academic entitlement—i.e., the belief that they deserve academic success regardless of performance. The shortened 17-item version was adopted from Hammoudi Halat et al. (1) who applied and validated the tool in a multi-national study among pharmacy students in the Arab world.

The questionnaire was translated into Arabic using the back-translation method to ensure both linguistic and conceptual equivalence. A pilot test was conducted with five students to ensure clarity, resulting in minor wording refinements. These pilot data were excluded from the final analysis.

Internal consistency was assessed using Cronbach’s alpha and yielded a value of 0.86, indicating acceptable reliability. The final questionnaire was distributed online via Google Forms.

### Data analysis

All data were coded and analysed using SPSS version 26.0 for Mac. Descriptive statistics, including frequencies, percentages, mean, and SD were used to summarize responses. Cronbach’s alpha was calculated to assess the reliability of the questionnaire. Independent sample *t*-test and ANOVA were used to measure the relationship between the AE score and independent variables including age, gender, and GPA. Cronbach’s alpha was used to assess the reliability (internal consistency) of the AE scale. Level of significance was set at *p* < 0.05.

### Ethics approval

Ethics approval was granted by the Ethics committee at King Khalid University, ECM#2024–3136.

## Results

A total of 276 students participated in the study and completed the online questionnaire. The demographic data reveals that the majority of participants fall within the age range of 21–26 years, with 35.1% aged 21–23 and 31.1% aged 24–26. A smaller proportion of participants are younger (18–20 years, 24.3%) or older than 26 years (8.7%), indicating that the sample primarily consists of individuals at a typical academic age. In terms of gender, the population is predominantly female, making up 79% of the sample, while males represent only 21%. Regarding academic performance, most participants demonstrate strong academic achievement, with 39.9% reporting a GPA between 3.75 and 4.49, and 38.4% achieving a GPA above 4.5. Only a small fraction of the sample has lower GPAs, with 17% falling between 2.75 and 3.74, 4% between 2.00 and 2.74, and just 0.7% below 2. Overall, the sample represents a predominantly high-performing, female-majority cohort within a standard academic age range Table 1.

**TABLE 1:** Demographics.

Factor	Number	%
**Age**		
18–20	67	24.3
21–23	97	35.1
24–26	88	31.1
> 26	24	8.7
**Gender**		
Male	58	21
Female	218	79
**GPA**		
≤ 2.74	13	4.7
2.75–3.74	47	17
3.75–4.49	110	39.9
> 4.5	106	38.4

The data reflects various dimensions of academic entitlement (AE) among students, with varying levels of expectations across different components. Students tend to have a moderate to high sense of entitlement when it comes to rewards for effort, with a mean score of 7.4 (74.2%), indicating they believe they should be recognized for their efforts. Similarly, there is a moderate expectation for accommodations, with a mean of 6.52 (65.2%), suggesting that students feel they should receive some level of flexibility in academic settings. However, the lowest score is in the area of responsibility avoidance, with a mean of 4.7 (47%), implying that students do not strongly believe they should avoid academic responsibilities, though there is some variation in individual responses.

The data also reveals that students have a moderate to strong expectation of customer-oriented service, with a mean score of 6.7 (67%) for customer orientation and a higher mean of 9.25 (61.6%) for customer service expectations. This suggests that many students expect a more personalized and service-oriented experience in their academic environment, though the variability in responses (evidenced by higher standard deviations) indicates that not all students share this view to the same extent. Grade haggling, with a mean of 8.64 (57.6%), indicates that students feel entitled to negotiate for higher grades, suggesting a belief that they should have influence over their academic outcomes.

Finally, the highest mean score is found in general academic entitlement, with a mean of 10.42 (69.4%), reflecting a strong sense of entitlement among students regarding their academic experiences. This suggests that students feel they deserve favorable treatment, higher grades, and perhaps more support throughout their academic journey. Overall, the data shows that while students exhibit a significant sense of academic entitlement, particularly in areas like rewards for effort and customer service expectations, there is notable variability in how these attitudes are expressed among individuals Table 2.

**TABLE 2:** Scores of the seven components of academic entitlement (AE).

AE component	Mean	SD	Min	Max
Rewards for effort	7.4 (74.2%)	2.0	2	10
Accommodation	6.52 (65.2%)	2.0	2	10
Responsibility avoidance	4.7 (47%)	2.1	2	10
Customer orientation	6.7 (67%)	2.1	2	10
Customer service expectations	9.25 (61.6%)	2.8	3	15
Grade haggling	8.64 (57.6%)	2.7	3	15
General academic entitlement	10.42 (69.4%)	3.0	3	15

The Academic Entitlement (AE) score was analyzed across demographic and academic variables, including age, gender, and GPA. A statistically significant association was observed between AE scores and age (*p* = 0.032), while GPA (*p* = 0.422) and gender (*p* = 0.242) were not significantly associated with AE scores.

Younger participants (18–20 years) had the highest AE scores (57.2 ± 11.0), followed by 21–23 (52.9 ± 12.2), 24–26 (52.8 ± 11.4), and > 26 years (50.3 ± 12.6), indicating a trend toward lower entitlement among older participants.

For GPA, the lowest-performing group (≤ 2.74), which included the two respondents previously in the “<2” category, had an AE score of 50.8 ± 13.9, while students in higher GPA categories (2.75–3.74, 3.75–4.49, and > 4.5) had relatively similar scores. No statistically significant trend or association was observed across GPA levels Table 3.

**TABLE 3:** Factors associated with academic entitlement (AE) score for the study population (*N* = 276).

Factor	AE score	*P*-value
Age		
18–20	57.2 ± 11.0	0.032
21–23	52.9 ± 12.2	
24–26	52.8 ± 11.4	
> 26	50.3 ± 12.6	
Gender		
Male	54.8 ± 12.5	0.242
Female	53.4 ± 11.7	
GPA		
≤ 2.74	50.8 ± 13.9	0.422
2.75–3.74	51.7 ± 13.7	
3.75–4.49	53.9 ± 11.5	
> 4.5	54.6 ± 11	

Academic entitlement (AE) scores are presented as mean ± standard deviation. AE scores were not weighted by group size. The “< 2 GPA” category included only two respondents and was merged with the next lowest category (2.74) for analysis due to limited representation.

**TABLE 4:** Internal consistency of academic entitlement (AE) questionnaire domains.

Scale/subscale	Number of items	Cronbach alpha
Rewards for efforts	2	0.76
Accommodation	2	0.67
Responsibility avoidance	2	0.66
Customer orientation	2	0.67
Service expectation	3	0.75
Grade haggling	3	0.60
General AE	3	0.89
Overall	17	0.86

The academic entitlement (AE) scale and its seven subscales, including Rewards for effort, Accommodation, Responsibility avoidance, Customer orientation, Customer service expectation, Grade haggling, and General AE, all demonstrated acceptable levels of reliability. The overall AE scale showed a Cronbach’s alpha within the acceptable range (0.6–0.7), and each subscale exhibited very good internal consistency, indicating their robustness in capturing various dimensions of academic entitlement effectively Table 4.

Assessing the association between demographic variables and academic entitlement (AE) components revealed notable patterns. A one-way ANOVA was conducted to examine the relationship between age groups and AE constructs. The analysis indicated statistically significant differences in Grade Haggling (*F* = 4.15, *p* = 0.0068) and Total Academic Entitlement (*F* = 3.04, *p* = 0.0294) across age groups. *Post hoc* analysis showed that students aged 18–20 had the highest mean scores in both Grade Haggling (9.58) and Total AE (57.32), suggesting greater entitlement tendencies among younger students. Similarly, ANOVA results based on GPA categories (with GPA < 2 merged into GPA 2–2.74) revealed a significant difference in the Rewards for Effort component (*F* = 5.72, *p* = 0.0008), with students in the highest GPA group (GPA > 4.5) reporting the highest mean score (7.84). No significant GPA-related differences were observed in the other AE components.

In contrast, gender-based comparisons using independent *t*-tests revealed a statistically significant difference only in the Responsibility Avoidance component (*t* = 3.84, *p* = 0.0002), indicating that male and female students differed in their tendency to avoid academic responsibilities. No significant gender differences were detected in the remaining components: Rewards for Effort, Accommodation, Customer Orientation, Customer Service Expectation, or Grade Haggling.

## Discussion

The study primarily aimed to assess academic entitlement (AE) among pharmacy students at a Saudi university, with a secondary aim to examine its significant associations with gender, age, and GPA. The demographic variables of age, gender, and GPA were selected based on their frequent associations with academic attitudes and behaviors in the literature. Previous studies have identified these factors as potential influencers of academic entitlement, making them relevant for the current analysis.

The findings of this study are consistent with previous research on AE in health-related education. For example, AE was found to be prevalent among pharmacy students in the Arab world, particularly among younger students—a pattern also reflected in our results, where students aged 18–20 demonstrated the highest AE scores compared to older age groups.

When comparing our results to those of the same study, the patterns of AE across the seven subdomains were largely consistent. Both studies reported similarly high scores for Rewards for Effort (74.2% vs. 76.4%) and General AE (69.4% vs. 70.4%), suggesting that students commonly expect academic rewards based on perceived effort and maintain a general sense of deservingness. Responsibility Avoidance was the lowest scoring domain in both studies (47% vs. 44.2%), indicating some level of student accountability. Notably, our cohort reported slightly lower scores in Customer Orientation (67% vs. 75.6%) and Customer Service Expectations (61.6% vs. 72.6%), which may reflect cultural or institutional differences in how students perceive their relationship with faculty. These comparable trends support the validity of the AE construct and highlight the utility of the questionnaire in capturing entitlement attitudes across pharmacy education settings in the Arab region (1).

Further analysis showed our study results are similar in many ways to those reported in the multi-national study on AE among pharmacy students in the Arab world by Hammoudi and colleagues. Both studies found that students felt strongly entitled in the Rewards for Effort domain. In our study, this was especially true for students with higher GPAs, while the previous study reported general agreement with this domain across all students. We also found that younger students (ages 18–20) had the highest scores in both Grade Haggling and overall AE. This supports the previous study’s finding that entitlement decreases as students progress through their program, shown by a negative link between year of study and AE. Regarding gender, our study showed a significant difference only in the Responsibility Avoidance domain, where males scored higher. In contrast, the previous study did not find any significant gender difference in AE, but it did report that female students had higher professionalism scores. These findings may point to cultural differences and underscore the need for further research to explore how gender-specific factors influence responsibility and entitlement perceptions in academic settings. Overall, both studies agree that GPA and academic maturity are stronger predictors of AE than gender (1).

Additionally, our results align with previous studies reporting no significant association between academic entitlement (AE) and GPA among pharmacy students. Jeffres and colleagues emphasized that AE attitudes can negatively affect academic performance and professional preparedness. Our study builds on this by demonstrating that high-achieving students (GPA > 4.5) tend to exhibit elevated scores in the “Rewards for Effort” domain. This finding suggests that academically successful students may develop a stronger expectation for recognition. This challenges the traditional view that AE is predominantly associated with underperformance and highlights the need to address entitlement across the full spectrum of academic achievement (2).

The AE questionnaire used in this study is a psychometrically validated tool originally developed by Jackson et al. (25) and adapted by Hammoudi Halat et al. (1) to the Arab educational context. In our study, the tool demonstrated strong internal consistency (Cronbach’s alpha = 0.86), supporting its appropriateness for assessing AE among pharmacy students.

Significant variations were observed across AE subdomains. The Rewards for Effort subdomain scored the highest (74.2%), indicating that students have high expectations for rewards based on their effort. In contrast, the Responsibility Avoidance subdomain scored lower (47%), reflecting varying levels of personal accountability. These findings suggest that academic culture may influence perceptions of entitlement, highlighting the need to review and evaluate methods in pharmacy education.

The results highlight the need for educational institutions to address AE among younger pharmacy students, particularly those aged 18–20, as AE can have detrimental effects on students’ academic performance and professional preparedness. To mitigate AE, it is essential for institutions to implement strategies that promote personal responsibility and professional behavior. Key strategies include promoting accountability, emphasizing effort over entitlement, and fostering a culture of professional responsibility. Faculty can implement interventions such as clearer communication of expectations and active learning methods to better prepare students for the demands of pharmacy practice. Additionally, the study found a significant association between AE and GPA, indicating that high-achieving students (GPA > 4.5) also exhibit entitlement behaviors, particularly in the Rewards for Effort domain. This highlights the need for a balance between recognizing student effort and maintaining rigorous academic standards, ensuring that students develop the skills necessary for success in pharmacy practice.

This study expands the understanding of AE in pharmacy education, particularly in Saudi Arabia. While AE has typically been linked to lower academic performance, our findings show that high-achieving students also exhibit entitlement behaviors. This challenges the traditional view of AE and highlights the need to examine entitlement across all levels of academic achievement.

### Limitations of the study

This study has several limitations. First, its cross-sectional design restricts the ability to draw causal inferences between academic entitlement (AE) and demographic variables such as age and GPA. Second, the sample was drawn from a single university in Saudi Arabia, and access to demographic data for the entire student population was not available, limiting our ability to assess representativeness and potential sampling bias. Additionally, the year of study was not collected from participants, which prevented analysis of AE trends across different stages of the academic program. This omission limits insights into how AE might evolve over time within the Pharm D curriculum.

The use of self-reported data also introduces the possibility of social desirability bias, where participants may have over- or under-reported entitlement-related attitudes or behaviors. While in-person recruitment improved the response rate, it may have introduced selection bias by disproportionately including students who were more frequently present on campus.

### Suggestions for future research

This study represents a foundational step in exploring academic entitlement among pharmacy students in Saudi Arabia. As such, it should be viewed as groundwork that establishes a basis for future investigations. The findings generate important hypotheses that warrant further exploration through longitudinal research. Specifically, it would be valuable to repeat this study at the same institution using the same methodology to determine whether students’ attitudes evolve as they progress through the Pharm D program. For instance, tracking first-year students into their third or fourth year could reveal whether their perceptions of academic entitlement shift with increased academic maturity, exposure to clinical training, or curricular transitions. Such longitudinal insights would enhance understanding of the developmental nature of academic entitlement in health education.

## Conclusion

The primary objective of this study was to assess academic entitlement (AE) among pharmacy students at a Saudi university and examine its significant associations with age, GPA, and gender. Additionally, the study explored the impact of AE on students’ academic performance. The findings revealed that younger students, particularly those aged 18–20, demonstrated the highest AE levels, while older students exhibited lower AE scores. Moreover, high-achieving students (GPA > 4.5) reported higher rewards for effort scores, while lower-performing students showed reduced perceptions of entitlement.

## Data Availability

The original contributions presented in this study are included in this article/supplementary material, further inquiries can be directed to the corresponding author.
